# Cerebral toxoplasmosis in children and adolescents from “Dr. Victor Babeş” Hospital pediatric HIV-cohort

**DOI:** 10.1186/1471-2334-14-S4-O26

**Published:** 2014-05-29

**Authors:** Sebastian Smâdu, Roxana Rădoi, Eugenia Ungureanu, Cristiana Oprea, Simona Tetradov, Mihaly Enyedi, Dan Duiculescu, Luminița Ene

**Affiliations:** 1 Clinical Hospital of Infectious and Tropical Diseases “Dr. Victor Babeş”, Bucharest, Romania; 2 Anatomy Discipline, Department of Morphological Sciences, Carol Davila University of Medicine and Pharmacy, Bucharest, Romania

## 

Cerebral toxoplasmosis (Toxo) is rarely described in children. The Romanian pediatric cohort consists of children that have been infected parenterally in the late ’80s. We aimed to describe the prevalence, clinical findings and outcome of Toxo in children and adolescents from the Romanian Pediatric Cohort, that have been diagnosed in the “Dr. Victor Babeş” Hospital, one of the main reference centers for HIV from Romania

Cerebral toxoplasmosis was diagnosed based on CDC case definition (presumptive and definitive diagnosis). We reviewed retrospectively the charts of all 29 children diagnosed with Toxo starting 1996, recording the demographic, HIV markers, antiretroviral treatment, clinical and neuroimaging data, treatment and outcome of Toxo.

The prevalence of Toxo was 4.8% from 604 patients followed in the Hospital. Out of 29 patients diagnosed with Toxo, 19 were girls and 28 had parenterally and 1 had vertically acquired HIV. The mean age at HIV diagnosis was 11.5±6.5 years, and 15.6±5.3 years at Toxo diagnosis. In 10 patients HIV was diagnosed concomitantly with Toxo. At onset 89.7% patients had focal neurological signs and 62.1% had headache. Median CD4 count was 60 (95% CI for median 27-95) lf/cmm. 28 patients had positive *T. gondii* IgG antibodies in plasma and/or cerebrospinal fluid. Only 6 patients had treatment with cotrimoxazole, most of them being treated with pyrimethamine/sulphadoxine combination and alternatively with atovaquone. 69% of patients had any adverse reactions to Toxo treatment. Most adverse reactions were cutaneous in 10 patients (5 severe) and anemia in 7 patients. Nine patients had antiretroviral therapy (ART) before Toxo diagnosis, out of them 1 had mono, 1 dual therapy, 6 were failing cART and one patient had immune reconstitution syndrome. Ten patients (34.5%) died. The median survival time was 117 months. In univariate analysis survival was correlated with shorter time from onset to admission (p=0.04) while on multivariate analysis diagnosis in the post-cART period was the only factor associated with longer survival (p=0.03). 14 of 19 patients recovered with sequels, most of them motor deficits.

Cerebral toxoplasmosis in this particular pediatric cohort shared common features with that reported in adults pertaining to prevalence in pre-cART period and pathogenic mechanism. Survival was associated with a more rapid diagnosis of cerebral toxoplasmosis and with access to cART.

